# Long-Term Sustainability of Water Cellars in Traditional Chinese Villages: Factors Influencing Continuous Use and Effective Water Management Initiatives

**DOI:** 10.3390/ijerph18094394

**Published:** 2021-04-21

**Authors:** Weinan Zhou, Kunihiko Matsumoto, Masanori Sawaki

**Affiliations:** Graduate School of Engineering, Osaka University, 2-1 Yamadaoka, Suita, Osaka 565-0871, Japan; matsumoto@see.eng.osaka-u.ac.jp (K.M.); sawaki@see.eng.osaka-u.ac.jp (M.S.)

**Keywords:** traditional rainwater harvesting system, traditional village, drinking water resource, water cellar, water cistern, renovation, sustainability

## Abstract

Traditional rainwater harvesting systems have seen a shift of emphasis in recent years. While recognizing its social, economic and environmental contributions, sustainable use in a modern context can be vulnerable. Through a case study, this study focuses on the long-term sustainability of water cellars in traditional villages if reliable piped water is introduced. The aim is to discern the factors and renovation methods that influence residents’ willingness to continue using these water cellars. The results show that the overall willingness to use them is very low. However, regardless of their continued use or non-use, only a few residents would landfill them. Most residents were interested in their renovation, especially regarding simplifying rainwater harvesting methods. In addition, the management time for rainwater harvesting and heritage identity is positively correlated with the willingness for sustainable use; conversely, the identification of the environmental contribution has no positive correlation. Given these findings, we propose carrying out effective renovation that changes the rainwater catchment surface to roofs and increases residents’ awareness that water cellars can only be heritage if they are in use. By defining the long-term sustainability of a water cellar, this study shows how a quantitative approach focusing on heritage users can offer important insights into a constructive evolution rather than a destructive reconstruction under the influence of modernization. Finally, this study provides planners and water resource managers with effective, sustainable management practices for water cellars as well as similar systems in a historical context.

## 1. Introduction

In many academic and practical fields today, traditional rainwater harvesting systems are rich sources through which researchers and planners interpret indigenous water culture, alleviate water distribution stress, build cultural landscapes, and avert hazards. For example, in the following cases, their benefits in a modern context have been increasingly recognized. Regarding widely built water wells, during the Great Hanshin Earthquake (1995) and the Great East Japan Earthquake (2011), their reliable function of ensuring safe water for daily life was recognized [1]. For larger systems, such as stepwells, their contributions to contemporary community cohesion [2] and distinctive tourism value [3,4] have been highlighted. For qanats that integrate with the settlement, their cultural landscape potential and integration with the urban environment are increasingly on the agenda [5,6]. However, many complex and delicate issues are pending, such as the promotion of disaster prevention registration systems for privately owned water wells [7]. At the same time, the revitalization of stepwells and qanats is always limited by groundwater depletion [8].

**Figure 1:**
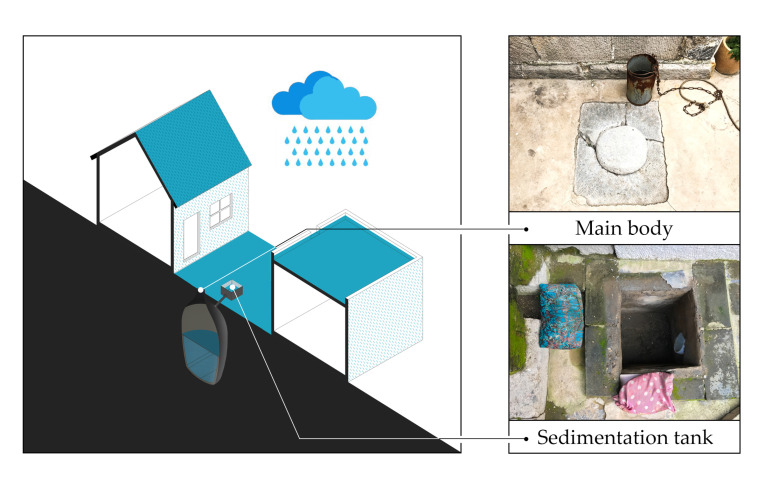
Rainwater harvesting system of water cellars and photos of their structure.

This study focuses on an indigenous rainwater harvesting system called a water cellar, which operates on a small scale in terms of rainwater catchment, storage, and capital investment [9,10]. To maximize the collection of adequate rainwater, the roofs (in the case of flat roofs, the drainage openings are generally oriented toward the yard) and the ground both serve as rainwater catchment surfaces (Figure 1). The rainwater converges in a sedimentation tank (some of the sediment from the roofs and ground remains at the bottom of the tank) and enters the storage body through a water inlet pipe. They are ubiquitously employed in many ancient settlements in Shanxi and Shaanxi, China, where rainfall is an uneven event and stops during dry periods. It is thanks to these water cellars that these settlements have survived. However, in the contemporary context, water cellars present many drawbacks, such as complex maintenance during the rainwater harvesting phase (e.g., when it rains, residents need to block the yard’s drains to enable more rainwater to enter the sedimentation tank [11]) and poor water quality [12]. However, the major problem may be the unstable water volume. Some deficiencies, such as poor water quality, can be addressed with water purifiers, but no water cellars, regardless of how perfectly renovated, can overcome drought [13]. The natural vulnerability of uses dictates that water cellars cannot serve as the dominant or sole source of water in modern society.

Sadly, most of these villages have no access to piped water systems even today because of their isolated location and underdeveloped economic conditions. Although this infrastructural decay has contributed to the authenticity of water cellar use, it comes at the cost of depriving residents of a modern form of water consumption. In 2012, China started a systematic investigation and registration of traditional villages for conservation. Starting in 2014, all registered traditional villages received CNY three million in conservation funds from China Central Finance. As demanded, parts of this funding could be prioritized for infrastructure development in some traditional villages that fail to ensure basic residential conditions; the remainder was to be invested in historic buildings and other conservation activities. Water cellars embody essential local water use cultures of traditional villages [11,14]. Furthermore, they can be a significant natural water resource if managed, as usual, considering a certain water supply capacity. Many studies recommend modernizing existing knowledge on rainwater harvesting to benefit water-scarce human settlements [15]. However, water cellars must be judiciously leveraged. In particular, it is important to balance the merits (for instance, convenience of use) and demerits (for instance, disruption to the cultural significance) of the renovation. Moreover, because water cellars are privately used, residents’ attitudes need to be incorporated into the planning and design. Therefore, with the arrival of piped water, the long-term sustainability of water cellars has become an important issue.

The concept of sustainability, emerging at the end of the last century, has evolved from a simple environmental sphere to include economic, social, and cultural aspects [16]. A classic definition regarding sustainability and conservation comes from Rodwell [17], which can be summarized as respecting the evolution of cultural heritage by providing minimal outside intervention. However, being a catchword, “sustainability” signifies different things to different people [17]; it varies with the object and focus range. In a study by Van Meter and colleagues [18], the researchers proposed a coupled natural and human framework for rainwater harvesting systems with a sustainability that could expand the focus from the scale of the villages to that of the basin, where more tangible social and economic benefits can be seen. Some scholars think that the conservation of qanats should go beyond the preservation of an ancient technology and requires a deeper understanding of the multiple roles of these structures in territories, society, life, and culture [19]. In studies focusing on Xinjiang qanats [20,21], researchers show that the sustainability of qanats requires integration with rural revitalization to achieve social, economic, and cultural complementarity. Similarly, in another study focusing on Iranian qanats [22], the researchers proposed that the traditional wisdom knowledge of qanats needs to be integrated into the scientific social–ecological system of the city to move toward sustainable water resource planning. At the same time, the sustainable relationship between urban expansion and these traditional water systems is gaining attention [23,24,25]. Very recently, it has been proposed that the sustainability of such projects could be attained through the perspective of cultural landscapes, not only for qanats [6] but also for all types of historical water systems, using an integrated view [26]. It has, however, been established that the sustainable connotations of traditional rainwater harvesting systems have gone beyond the systems themselves and should be multi-dimensional and involve innovation, such as through relationships with human beings, urbanization, and various urban systems. In this study, we define the “long-term sustainability of a water cellar” as “meeting the present needs of the users (property owners) while providing constructive evolution in harmony with water cellars’ fabric, cultural identity, and historical environment in a way that ensures continuity of use as a priority”.

Unfortunately, little attention has been paid by society and academia to the long-term sustainability of this endangered rainwater management system, which is a collaboration between nature and humans, and particularly residents’ evaluations and perceptions. Besides the early fieldwork-based historical research [14], recent studies have mainly focused on the flood prevention function of water cellars using simulated data [27] and their operation mechanisms [11]. Furthermore, some studies have explored the renovation of water cellars, such as improving water quality by adding water purifiers to the storage body [12] and increasing storage capacity by connecting water cellars and water-logging pools [28]. Unfortunately, these renovations do not consider whether residents are still willing to use them continuously and whether they would want renovations if piped water were available. The loss of focus on user opinions is the same as in other systems, such as ponds, stepwells, and qanats. Being mostly publicly owned, their sustainability is realized more through management optimization by the community [29,30] rather than through the attitudes of residents. Another major reason for the neglect is that, as mentioned earlier, they face more serious abandonment problems due to groundwater deficiencies. Thus, research on groundwater compensation [8,31,32] and macro groundwater regulation [33,34,35] is now the center of attention.

Recently, a few researchers have also drawn attention to public awareness. In a study by Canavas [36], the importance of public awareness in transforming qanats into a national heritage was emphasized. Feng believed that after the introduction of piped water, the ancient regulations for water use and routine maintenance of qanats could be seriously undermined [37]. In response to the phenomenon that, although they were facing the same worsening water crisis, some farmers were profligate in their use of water but others were parsimonious, Yazdanpanah and colleagues [38] proposed that modern technology could be adapted along the lines of the traditional qanat socio-technical system to provide water as a common good (egalitarianism) rather than as it is currently, as a public good (hierarchy) or private good (individualism). Quite recently, Qasim and colleagues, through a questionnaire method, clarified that membership on a qanat committee, land holding, and land tenure security strongly affect willingness to pay for qanat rehabilitation [39]; however, a specific renovation solution was not involved. Kowkabi incorporated the attitudes of study participants into the planning and evaluation of the qanat revitalization route through a map and interview analysis [40]. While these studies have observed the importance of heritage identity, the traditional water distribution model, and the impact of modernization on residents’ perceptions of use, quantitative surveys are limited in being able to provide a defined reference for renovation and water management. Thus, it is urgent that quantitative methods be used to objectively understand residents’ perceptions of the renovation and conservation of traditional rainwater harvesting systems, regardless of whether they are privately or publicly owned.

This study aims to understand water cellars’ long-term sustainability in traditional villages if reliable piped water is introduced and to provide effective water management initiatives for planners and water resource managers. Therefore, through a case study, the following are examined: (1) the satisfaction of daily use, identification of environmental contribution, and heritage identity on the willingness for continuous use; and (2) the impact of the renovation solutions accepted by the residents on its heritage authenticity. Not limited to water cellars, the quantitative approach focusing on heritage users in this study can also be used for understanding the long-term sustainability of similar systems.

## 2. Materials and Methods

**Figure 2:**
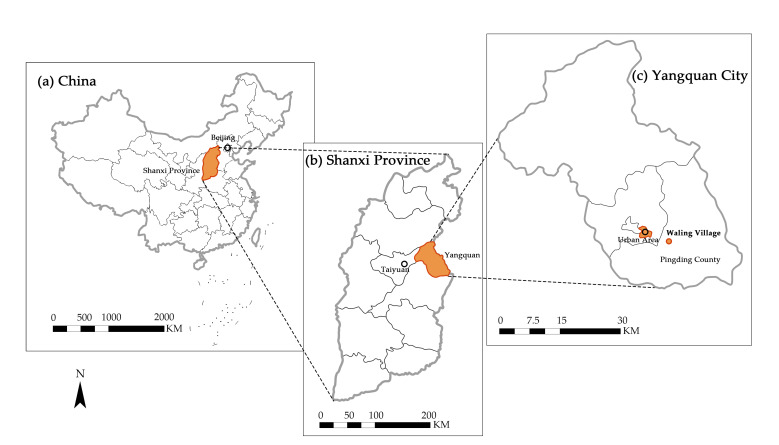
Research area.

This study used 56 questionnaires (one questionnaire for one person per household) collected from a typical traditional village, Waling in Yangquan City, Shanxi, China (Figure 2), where each household owns a water cellar for domestic water consumption. Yangquan City is located on the eastern edge of the Loess Plateau, the western part of the Taihang Mountains, where the landscape is dominated by mountains and hills. The economy of Yangquan City is based on coal mining, although despite the rich historical tourism resources of settlements, the tourism industry has not yet been developed due to the inconvenience of transportation and inadequate public utilities. According to its conservation and development plan, Waling village is surrounded by mountains, with around 70 peaks above 1000 m in elevation, and the residents’ ancestors settled here as late as the Chinese Song Dynasty. The tradition of water cellar construction can be traced to the Ming Dynasty, according to local historical records of Waling village (unpublished). The underdeveloped economic and transportation conditions made the villages suffer from population loss very early. At present, there are hardly any young or even middle-aged people living in the village, because they have mostly bought houses at affordable prices in downtown Yangquan or in Pingding County, where daily living needs and children’s schooling can be accommodated. The questionnaire survey was conducted from 15 November to 19 November 2020. The investigators visited households, delivered the questionnaires to the respondents with consent, explained the instructions for filling them out clearly, and then retrieved them after they were completed; for some residents who could not read, the investigators asked each question and recorded their answers one by one. The number of permanent households in the village was approximately 80, and the survey response rate was approximately 70%. The village was selected because of its well-preserved historical environment since it was registered as a national traditional village in 2013, it had a long history of water cellar construction, and it had no piped water.

Besides demographic information (Table 1), the questionnaire devised in this case study consisted of five main sections: willingness to continue to use (if reliable piped water is introduced), satisfaction evaluation of daily use, identification of environmental contribution, heritage identity, and possible utilization patterns (if reliable piped water is introduced). The selection of satisfaction evaluation factors aimed at a completeness of evaluation and considered both the inner (water factors such as water quantity and quality) and the outer (structure and appearance) features of water cellars. Most importantly, considering that the evaluation of usage defects was the focus of this study, it was necessary to check whether the daily use of water cellars is a physical and time burden for the residents. Overall, the assessment was divided into four parts: evaluation of water bodies, burden of daily use (water lifting method and management time for rainwater harvesting), evaluation of structure and appearance, and overall evaluation. The value of this environmental contribution was determined by referring to our preliminary basic research [11], which examined the interaction between humans and the village environment brought about by the use of water cellars. Finally, regarding the determination of possible usage patterns, we refer to the current market design of modern household rainwater harvesting systems [41] that use roofs as rainwater catchment surfaces; at the same time, we predicted some participant behaviors such as maintaining the original water cellar but not using it or landfilling it. This study is structured in two parts. In the first section, by testing the correlation between the willingness for continuous use and three influencing factors (satisfaction of daily use, identification of the environmental contribution, and heritage identity), the study can define the technical improvements required and the authentic state of core value identification and heritage understanding of water cellars. The second section outlines acceptable water consumption activities and treatments to ascertain the direction of technological renovation of water cellars. In particular, among these treatments, some solutions may be damaging to the heritage authenticity of water cellars. On this basis, this study examines how to balance technological renovation and heritage conservation to reflect the long-term sustainability of water cellars. Notably, despite variation in the construction techniques of water cellars between regions characterized by locally available materials, customs, and tradition, similarities are shared in Waling village. Furthermore, apart from the lack of access to piped water systems, the technological modernization of water cellars in this village is limited to applying concrete for the impermeable layer (Table 2).

## 3. Results

### 3.1. Influencing Factors for the Willingness for Continuous Use

#### 3.1.1. Willingness for Continuous Use If Reliable Piped Water Is Introduced

**Figure 3:**
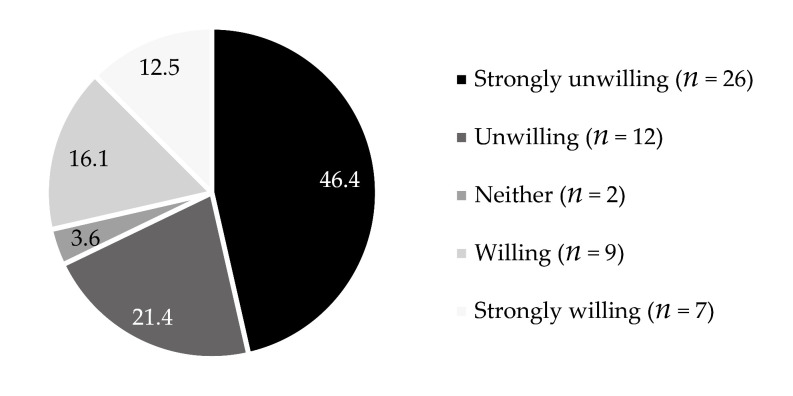
Willingness to use water cellars continuously (*n* = 56).

Figure 3 shows residents’ willingness to continue using water cellars. The percentage of those who did not want to use them (67.8%) was much higher than that of those who wanted to (28.7%). Hence, the results are not related to the demographic data of the respondents (gender: *p* = 0.485; group: *p* = 0.825; occupation: *p* = 0.955; family type: *p* = 0.718) or the characteristics of the water cellars (location: *p* = 0.560; age: *p* = 0.816; impermeable layer: *p* = 0.510) (*p*-value indicates difference between attributes).

#### 3.1.2. Influencing Factor One: Satisfaction of Everyday Use

**Figure 4:**
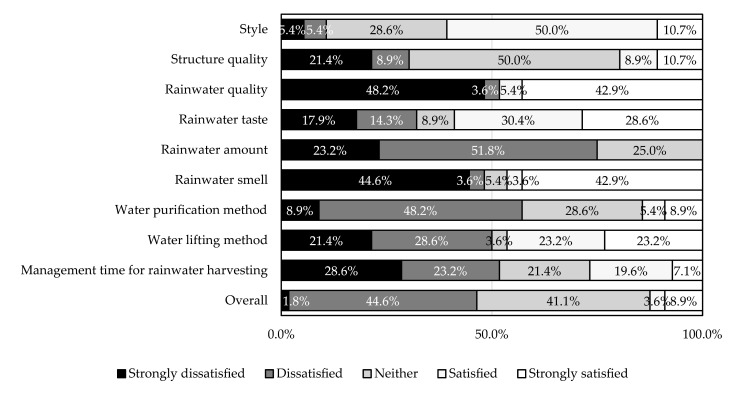
Satisfaction evaluation of everyday use (*n* = 56).

The results of the satisfaction evaluation for everyday use are shown in Figure 4. The dissatisfaction ratios of more than one-half were related to rainwater quality (51.8%), rainwater amount (75.0%), water purification method (57.1%), water lifting method (50.0%), and management time for rainwater harvesting (51.8%). Conversely, over one-half of the satisfaction was related to style (60.7%) and rainwater taste (59.0%). Overall satisfaction was extremely low (satisfaction: 12.5% versus dissatisfaction: 46.4%).

**Figure 5:**
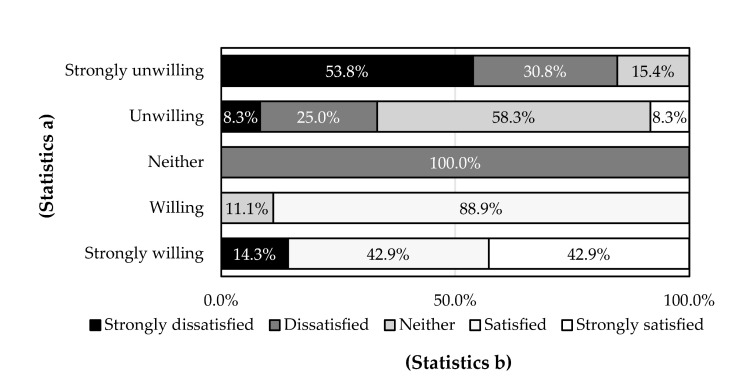
Cross analysis of the willingness for continuous use (Statistics a) with satisfaction of management time for rainwater harvesting (Statistics b) (*p* = 0.000).

When the willingness for continuous use with satisfaction evaluation was cross analyzed, only the satisfaction with management time for rainwater harvesting was strongly related to the willingness for continuous use (*p* < 0.001) (Figure 5). Clearly, they were positively correlated. The respondents who were strongly unwilling to use water cellars continuously had the most negative evaluation of the management time (84.6%) and no positive evaluation; those who were unwilling to use water cellars continuously had a slightly positive evaluation (8.3%). Meanwhile, most respondents who were willing to use water cellars continuously made positive evaluations of the management time (88.9%), which is almost equal to the positive evaluation made by those strongly willing (85.8%).

#### 3.1.3. Influencing Factor Two: Identification of Environmental Contribution

**Figure 6:**
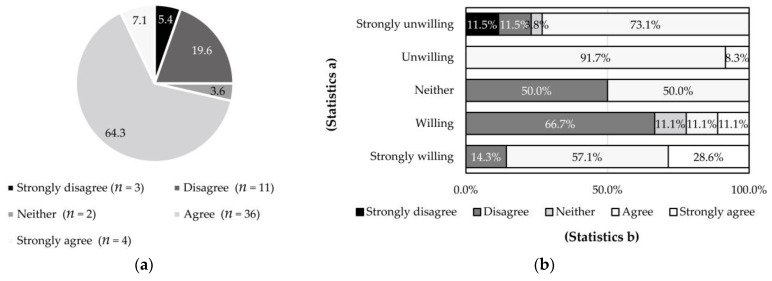
Identification of the environmental contribution of water cellars and its cross-analysis with the willingness for continuous use: (**a**) identification of the environmental contribution of water cellars (keeping yards, roads, and public spaces clean); (**b**) (statistics b) cross-analyzed with the willingness for continuous use (statistics a) (*p* = 0.008).

More respondents were convinced of the environmental contribution of water cellars (71.4%) (Figure 6a). However, this was not positively correlated with the willingness for continuous use (*p* < 0.01) (Figure 6b). Nevertheless, most respondents who were strongly unwilling to use water cellars continuously acknowledged their environmental contribution (73.1%), as did most respondents who were unwilling to use them. However, most respondents who were willing to use water cellars continuously had the highest percentage of disagreement with the environmental contribution (66.7%); meanwhile, even a few of those very willing to use the water cellar did not agree (14.3%).

#### 3.1.4. Influencing Factor Three: Heritage Identity

**Figure 7:**
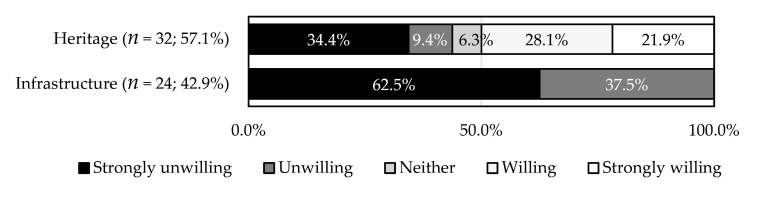
Cross analysis of the willingness for continuous use with heritage identity (*p* = 0.000).

The respondents more often identified water cellars as heritage (57.1%) than infrastructure (42.9%) (Figure 7). This has no apparent correlation with their characteristics (age: *p* = 0.654; location: *p* = 0.280; impermeable layer: *p* = 0.385). Of the respondents who identified water cellars as heritage, 50.0% preferred using them continuously; however, a large percentage of the respondents did not prefer this, even though they identified water cellars as heritage (43.8%) (*p* < 0.001) (Figure 7). This implies variability in the residents’ interpretations of heritage. Furthermore, none of the respondents who identified water cellars as infrastructure preferred to use them continuously.

### 3.2. Possible Utilization Patterns

**Figure 8:**
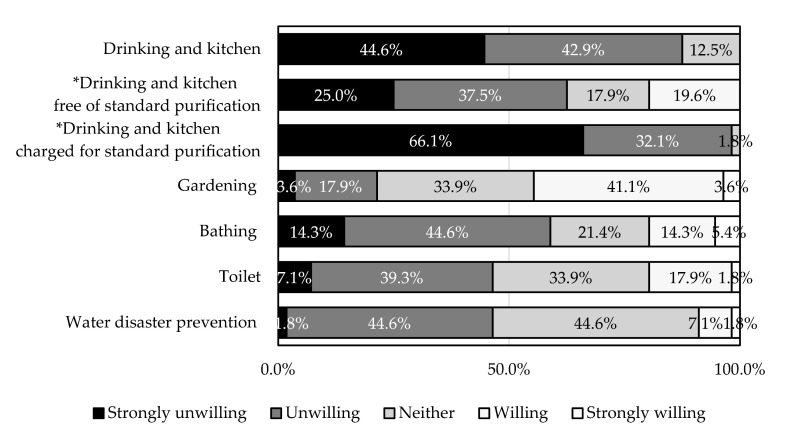
Acceptable water consumption activities of water cellars complementary to piped water (*n* = 56). * Hypothetical conditions.

Figure 8 shows acceptable water consumption activities of water cellars complementary to piped water. More than half of the unacceptable water consumption activities were drinking and kitchen use (87.5%), drinking and kitchen use free of standard purification (62.5%), drinking and kitchen use charged for standard purification (98.2%), and bathing (58.9%). Conversely, the percentage of acceptable activities did not exceed half of the total: drinking and kitchen use if it is free of standard purification (19.6%), gardening (44.7%), bathing (19.7%), toilet use (19.7%), and water disaster prevention (8.9%). Meanwhile, drinking and kitchen as well as charged for standard purification witnessed no acceptability.

**Figure 9:**
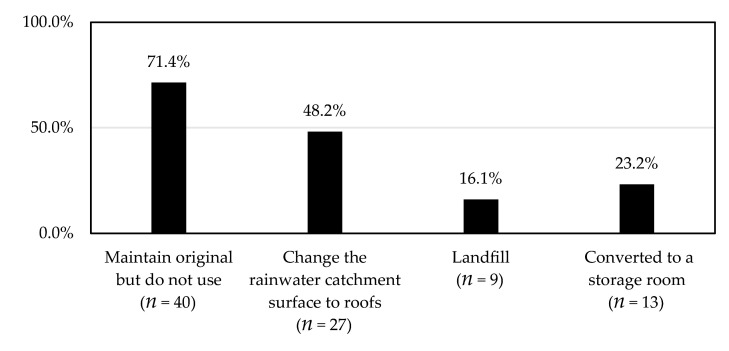
Acceptable treatment of water cellars (more than one option can be selected) (*n* = 56).

Figure 9 shows the acceptable treatment of water cellars by the respondents. The results are not strongly related to the willingness for continuous use. Maintaining the original water cellar but not using it was ranked the highest (71.4%). This was followed by the time-saving design practice of changing the rainwater catchment surface to roofs (48.2%). Landfilling accounted for the lowest percentage (16.1%). Moreover, a few respondents were also interested in functional conversion (converted to a storage room: 23.2%).

## 4. Discussion

(1) The willingness to use water cellars sustainably, if reliable piped water was introduced, was fairly low (28.7%). This was not strongly related to the demographic data of the respondents and the characteristics of water cellars. Meanwhile, only a few respondents could accept landfilling (16.1%), and most intended to keep the original water cellar but did not intend to use it (71.4%). This suggests that respondents were not bored by the presence of the water cellar and were not very resistant to renovation or transformation, regardless of whether the rainwater catchment surface was changed to roofs (48.2%) or the water cellar was converted into a storage room (23.2%). The transformation of the storage space is not recommended, because it is costly and affects the yard space. Thus, most water cellars may become a physical monument under no planning or management intervention if reliable piped water is introduced. This is an important finding because physical destruction means that any later revival becomes imperative.

Moreover, the willingness to consume rainwater from water cellars as a supplement to piped water for specific water use activities was also low (all below 20%, except for gardening: 44.7%). None of the participants intended to continue drinking it, and kitchen activities and the provision of free purifiers did not make a big difference (19.6%). Thus, the renovation method of improving water quality by adding a water purifier to the storage body [12] may not be acceptable to the residents. The same is also difficult to practice because of the high cost of purifiers. Therefore, it is not advisable to include a chemical purification component in water cellars’ structure or provide water purifiers to the residents. This result suggests that the original water supply function optimization of water cellars should be dedicated to a certain function other than drinking and kitchen use.

However, because heavy droughts can sometimes occur, it is unwise to rely entirely on a water cellar for bathing or toilet use. Both consume large amounts of water, and one cannot take the risk of disruption. Therefore, we suggest that the water cellar is best designed for gardening use, which also had the highest willingness (44.7%).

(2) While there were various satisfactory evaluations of the everyday use of water cellars, only the management time for rainwater harvesting was strongly related to the willingness for their continuous use. This suggests that their structure’s optimization should simplify the traditional way of using human power to liberate the residents from cumbersome management. Notably, changing the rainwater catchment surface to roofs, a convenient and low-cost structure optimization addressing this drawback, witnessed some expectations from the respondents (48.2%). From this perspective, this structural optimization of water cellars may be worth implementing to increase the willingness for continuous use.

If the rainwater catchment surface is reduced to roofs, the amount of rainwater stored each year will drastically decrease. This will inhibit the use of water cellars for bathing and toilets. Nevertheless, because we have defined that long-term sustainability should provide constructive evolution in harmony with water cellars’ fabric, cultural identity, and historical environment, this renovation needs to avoid the direct application of modern techniques and should inherit the original structure and advantages. First, the conveyance part demands the use of traditional materials, and its style cannot spoil the historical atmosphere. Secondly, considering that traditional structures’ integrity is an essential part of heritage conservation, the first flush system (a system diverting the first flow containing the sediments laying on roofs away) better reuses the original sedimentation tank structure (displayed in Figure 1), and the storage system necessitates the use of the original structure. Thirdly, there is no need to add a filtration system that is not included in the traditional structure because drinking and kitchen use is not expected. Finally, the overall cost needs to be controlled and can affect the residents’ acceptance, because the traditional construction method is low-cost. The literature on urban rainwater harvesting systems has also called attention to waterborne diseases due to a lack of maintenance knowledge [42] and economic constraints [43].

(3) In terms of the environmental contribution of water cellars, although most respondents agreed with it (71.4%), it was not positively correlated with the willingness for the water cellar’s continuous use. However, if the rainwater catchment surface turned into an automated roof catchment system (i.e., the ground was no longer a rainwater catchment surface), the long-established environmental relationship between water cellars and the village will be disrupted. However, despite the environmental contribution being compromised, the renovation is still accepted because the traditional structure and its advantages that encompass most of its cultural significance can be inherited.

(4) The heritage identity of water cellars is also closely related to the willingness for their continuous use. No respondents considered water cellars as a desired infrastructure for continued use. If water cellars are only considered as infrastructure, they have no utilization significance as far as the residents are concerned. However, many respondents did not prefer to use them even if they identified water cellars as heritage (43.8%). Thus, to increase the willingness for continuous use, one cannot simply propose reinforcing residents’ awareness of water cellars’ heritage attributes. Rather, the focus should be on the fact that only a water cellar that is used can conserve the authenticity of this everyday heritage.

## 5. Conclusions

This study has defined the long-term sustainability of a water cellar for the first time. From the perspective of the residents, this study reflects on how water cellars in a historical context can achieve a constructive evolution as opposed to a destructive reconstruction if reliable piped water is introduced. Overall, the study contributes to existing research on the long-term sustainability of traditional rainwater harvesting systems by providing evidence that their renovation programs and residents’ willingness are inextricably linked. In the presence of contradictions between renovations and the conservation of inheritance properties, this study provides an illustrative view that some heritage characteristics can be conceded for sustainable use, as long as the majority survive. Moreover, more traditional villages using water cellars or similar systems, albeit with different demographic characteristics, geographical locations, and social environments, are still awaiting investigation, and it is suggested that they be carried out by employing this research approach.

## Figures and Tables

**Table 1:** Demographic data of respondents from each household (*n* = 56).

Variable		Number (*n*)	Percentage (%)
Gender	Male	34	60.7
	Female	22	39.3
Age group	45–54 years old	2	3.6
	55–64 years old	3	5.4
	65–74 years old	28	50.0
	75–84 years old	23	41.1
Occupation	Farmer	53	94.6
	Unemployed	1	1.8
	Self-employed	2	3.6
Family type	Single	1	1.8
	Couple	36	64.3
	Couple and child	1	1.8
	Widowhood	18	32.1

**Table 2:** Characteristics of target water cellars from each household (*n* = 56).

Variable		Number (*n*)	Percentage (%)
Age	1920s	1	1.8
	1960s	5	8.9
	1970s	10	17.9
	1980s	18	32.1
	1990s	15	26.8
	2000s	7	12.5
Location	In the middle of yards	16	28.6
	Close to the door	13	23.2
	Close to the kitchen	10	17.9
	Close to the main house	7	12.5
	In public spaces	10	17.9
Impermeable layer	Clay	34	60.7
	Concrete	22	39.3
